# Medical Opinion and Movement

**Published:** 1911-09-30

**Authors:** 


### Medical Opinion and Movement.

### Disinfection by Thymolated Alcohol.

In the *Zentralblatt für Chirurgie* Drs. König and Hoffmann recommend the use of thymolated alcohol for disinfection of the skin over the region to be operated upon in preference to tincture of iodine. According to experiments carried out by them, an application of a solution of 1 part thymol to 100 parts alcohol at 60° so completely sterilises the skin of a rabbit without previous washing that a fragment of the skin placed in bouillon and incubated remains sterile indefinitely. If only 0.25 per cent. thymol solution be used, the bouillon remains clear for the first day but becomes cloudy after two or three days. In order to ensure complete sterilisation the authors use 5 per cent. thymol solution and apply it to the skin twice at an interval of a few minutes. The skin becomes red and there is a burning sensation immediately after the application; but there is no subsequent irritation or eczema, such as not infrequently results from the tincture of iodine. The authors have employed this method of disinfection in 130 cases, and the results have been entirely satisfactory, so that they are convinced that this thymol solution is at least equal to the iodine as a means of disinfection, and it has the advantage of not irritating or discolouring the skin, and is also much cheaper.

### Insufflation of the Colon.

The diagnostic value of gastric insufflation is already well recognised, but Dr. Walter A. Bastedo, of St. Luke's Hospital, New York, draws attention to the practice of insufflating the colon as a means of diagnosis in abdominal affections. He uses for this purpose a soft rubber sound, which is introduced into the rectum to the extent of about 30 centimetres, and through this the lower bowel is distended with air. By distension of the lower bowel the anatomical disposition of the sigmoid, transverse colon, and cæcum can be accurately gauged, and the transverse colon can be differentiated from the stomach. The muscular tone of the large intestine can be determined. If it is flaccid and atonic it can be distended enormously without inconvenience to the patient; otherwise it gives rise to colic pain or results in an expulsion of air by the rectum. The position of an occlusion of the lower bowel can be localised by this method, provided there is no marked tympanism already present. The author also points out that this insufflation divides the abdominal cavity into two distinct anterior and posterior regions and renders an organ or tumour of the anterior region more prominent, while it pushes backwards and conceals an organ or tumour of the posterior region, and in this way assists in the diagnosis of abdominal conditions. According to Dr. Bastedo, insufflation of the colon also is of assistance in the diagnosis of doubtful cases of appendicitis. The occurrence of acute pain or simply of a sensitiveness in the region of McBurney's point on insufflation is, according to his experience, diagnostic of inflammation of the appendix. He has had several cases in which this has been confirmed by subsequent operation; and, contrariwise, cases in which appendicitis has been suspected but this sign failed, the condition has completely cleared up without subsequent recurrence, or operation has disclosed a normal appendix.

### "Return Cases" of Diphtheria.

An interesting report on "return cases" of diphtheria at the Blegdam Hospital at Copenhagen is made by Dr. Sörensen in the *Hospitalstidende*. During the last twelve years 7,037 cases of diphtheria have been treated there. There were eighty-two "return cases"—that is to say, there were eighty-two fresh cases of diphtheria among members of their families after the patients had returned home, or a proportion of 1.16 per cent. Of the eighty-two patients who were the apparent cause of infection, eight-one had been examined bacteriologically, and only eight showed the presence of the diphtheria bacillus in the throat. Of the 7,037 diphtheria patients during the twelve years about seven hundred were carriers of the Löffler bacillus when they returned home, and yet these seven hundred patients only supplied eight "return cases," or 1.14 per cent.—that is, almost the same proportion as given above, 1.16 per cent. It appears, therefore, that the bacillus carriers are not more dangerous to their families than those ascertained by bacteriological examination to be exempt. In other words, according to these statistics, the bacteriological examination of the throat, upon which so much stress has been laid recently, would appear to have by no means the same value that has been attributed to it as a means of determining the infectibility of a patient after an attack of diphtheria. The author further states that the "return cases" were not specially severe, and there was not a single fatal case among them. This is, of course, contrary to the experience with scarlet fever, when the "return cases" are generally much more severe than the initial cases.

### Anteflexion and Uterine Myoma.

In the *Zentralblatt für Gynäkologie*, Dr. D. Von Ott puts forward his views in regard to the ætiology of uterine fibro-myomata. By following for several years patients affected with anteflexion of the uterus, he finds that a large proportion subsequently develop myomata, and, on the other hand, he has obtained from patients afflicted with fibromata accounts of symptoms strongly suggestive of a previous condition of anteflexion of the uterus, so that he is led to the conclusion that anteflexion is an important ætiological factor in the production of uterine fibro-myomata. It has been ascertained that not infrequently fibro-myomata take their origin from the muscular wall of the veins. Any irritation or super-activity in the venous circulation might, therefore, favour the development of such a tumour. The veins are naturally more affected by compression and elongation than the arteries, on account of the walls being thinner and less resistant. A condition of uterine cirrhosis results, as evidenced frequently by the premenstrual pain that the patients suffer. As a phophylactic measure, therefore, the anteflexion should be corrected. For this purpose the author advises excision of the cervix. By this means sterility may at the same time be cured. According to the statistics of the author, subsequent pregnancy has taken place in one-third of the cases operated upon by him.

### The Viability of Ascaris Ova.

Some curious points in the life history of the ova of *Ascaris lumbricoides* have been observed by Dr. R. S. Morris, who writes on this subject in the *Johns Hopkins Hospital Bulletin*. In the clinical laboratory of that hospital are preserved jars containing a thin suspension of fæcal material and parasitic ova. The dilution with formalin and water is so arranged that there is present 2 per cent. of the antiseptic. This strength was believed to be lethal to the ova of every parasite affecting human beings, and Dr. Morris's experience shows that this is true of. all except *Ascaris*. In January 1909, when demonstrating from one of these jars, he was astonished to find that many of the eggs contained actively motile embryos. How long the specimen had been on the laboratory shelf is unknown, but the embryos were still motile in May of this year—that is, twenty-nine months after they were first noticed to be alive. In December 1909 the author had an opportunity to test the accuracy of his observation, as a patient was admitted whose fæces swarmed with the ova of *Ascaris*. A specimen was put up in 2-per-cent. formalin, securely sealed in a jar of brown glass, and left on the laboratory shelf. Twice in 1910 search was made for motile embryos without result, but in January 1911 a few were found, and in May another examination showed an apparent increase of the number. The viability of these ova is, therefore, evidently very great.

### The Suturing of Kidney Incisions.

The interrupted catgut suture is probably that most affected by surgeons who have frequent occasion to explore and then to sew up the kidney, as it certainly seems to act better in practice than any other measure for securing repair and hæmostasis. In the *Annals of Surgery*, Moore and Corbett, after experimenting with various suture methods on rabbits, enunciate the following propositions: An operation upon the kidney always destroys some kidney substance. The actual section of the kidney does less harm than the suturing necessary to control hæmorrhage. Suture of the capsule alone is not sufficient to control hæmorrhage, and is therefore dangerous. Mattress sutures destroy a great deal of kidney substance, and the enlargement of the opposite kidney after a short time is due to congestion from overwork. The destruction of kidney substance extends far beyond the field of operation. The functional activity of the wounded kidney is somewhat reduced. Interrupted sutures transfixing the kidney at the pyramidal line and tied around the body of the kidney constitute the method of suture which does least damage to the kidney.

### The Treatment of Pneumonia.

In a vigorous article in the *Medical Record* Dr. Mitchell denounces most of the remedies which are in common usage when dealing with pneumonia cases. The logical treatment, according to this writer, is rest, support, and calcium. Absolute quiet, with interdiction of visitors, is no novelty; but the rest of the régime thus ordered is certainly rather new. The sole diet allowed is equal parts of milk and lime water, and the medicinal part of the treatment is 10 grains of calcium chloride every three hours; so the patients certainly get plenty of calcium one way and another. All other treatment is unsparingly castigated. Stimulants of all kinds, and alcohol in especial, are declared to be useless; expectorants, inhalations, and pulmonary antiseptics are the same. Cold sponging and cold air are to be avoided just as much as are antipyretics. Quinine and guaiacol are also warned against. The treatment of pain does not come under notice at all in this somewhat intolerant article, nor that of cough, nor that of constipation. The author apparently pins his faith to calcium, and calcium only, partly as the result of clinical experience, but more largely on a bacteriological basis.

### Pituitary Extract after Laparotomy.

To discover how far this drug, unaided by aperients, is able to restore peristaltic contractions, Mr. L. Bidwell tried its effect in twenty-one unselected laparotomies in the West London Hospital, and records the results in the *Clinical Journal*. In the first two cases the dose given was ½ c.c., but in the later cases this was increasd to 1 c.c. (for adults); the dosage was commenced about six hours after operation and repeated every four hours for eighteen doses. Each dose was given by intramuscular injection, which is said to avoid the pain consequent upon subcutaneous injection. Although careful antiseptic precautions were taken, it appears that local suppuration was encountered; but no unpleasant general effects were noted. Practically all the patients passed flatus soon after the treatment began, and abdominal distension or discomfort was conspicuously absent. Only three patients actually defæcated without the assistance of an enema; but it was unnecessary to give any aperient by the mouth. Mr. Bidwell advises as a routine practice after laparotomy the injection of 1 c.c. three times in the first twenty-four hours the first six hours after operation, and the others at the same intervals subsequently. Patients thus treated are, he says, more generally comfortable than those not so treated.

### The Determination of Sex.

Speculation as to the reasons that cause about half the babies born in the world to be boys and the other half to be girls, and attempts to forecast and measures devised to influence the sex of unborn children have been very numerous in all ages. The present century finds the problem as far from solution as ever, notwithstanding a large amount of ingenuity expended over its investigation. The latest hypothesis is one advanced by Mrs. David McConnel, of Stanford University, in the United States. She believes that a child conceived one or two days before, or one or two days after, menstruation, will be a female; but that those conceived at all other times will be males. The male parent has no influence on the determination of the sex, which is controlled entirely by the physical state of the ovum at the time of impregnation. In thus allotting to the maternal cell the entire responsibility for the sex of the fœtus, Mrs. McConnel is in agreement with several recent authorities on this subject. It may also be noted that some of her views are very similar to those of Oliver, published lately in the *Lancet*. Her speculation is interesting, and it may be hoped that it will be independently tested, though obviously the difficulty of obtaining positive evidence is great. Mrs. McConnel rejects all evidence unless the possibility of conception has been reduced to one occasion per menstrual cycle. She does not give much detail of the observations and experiments on which her views are founded, but states that every carefully carried out attempt thus to control the sex of a child has resulted as desired, when the conditions of health and physical development have been normal. She mentions a few of these successful attempts, and says she has records of many others. The hypothesis is tempting, but requires a good deal more substantiation by experiment than has yet been offered.

### Suicide Due to Hypersensitiveness.

The suicide recently of a girl owing to the fact that the presence of a birthmark had "preyed upon her mind," illustrates the pathetic influence which a physical deformity may have upon the mind. Children, unfortunately, are generally unable to appreciate the pain which may be inflicted by reference to a deformity. To them the deformity may provide reasons for indulging in remarks possibly considered to be witticisms, in the enjoyment of which they very likely think the one treated less kindly than themselves by nature ought to join. Thoughtless childhood may even find in a feature which presents nothing particularly noteworthy a source of harmful amusement. For example, a girl now grown to womanhood and possessed of personal beauty far above the average, used to be teased when young by her brothers on account of the delicacy and small size of her nose. Although there can scarcely have been any desire to inflict pain, the teasing apparently caused considerable disturbance of the child's peace of mind. The idea that she was afflicted with a serious blot upon her face took possession of her, and she used to pray nightly that if possible her affliction might be removed. Her trouble over a feature which she has no doubt learnt by now to be "a thing of beauty," is looked back upon after the lapse of years with some amusement. But it would probably have been otherwise had her nose really been sufficiently peculiar to attract unpleasant attention. Instead of growing up with a humorous bright nature, there might have been a shrinking hypersensitiveness which would have destroyed enjoyment of life. Consideration for the feelings of others in matters like these should occupy an important place in a child's education.

### The Treatment of Burns.

A short time ago an American surgeon, Dr. J. F. Alexander, was called upon to treat twenty-seven patients suffering from severe burns, sustained at the same time in a blazing house. All the burns were of the first, second, and third degrees, and all were severe. Even the distribution of the burns was somewhat uniform, as in nearly every patient there were burns of the face, arms, and chest. Wishing to test the relative values of some of the best known methods of treatment, Dr. Alexander, who sums up his experiences in the *International Journal of Surgery*, divided the cases into batches. Seven burns were treated with boric acid solution baths; five with gauze kept constantly soaked in picric acid solution; five with carron oil dressings; five with unguentine; and five with ichthyol. The boric acid baths were used for three hours at a time, with six hours' interval, at first; but it was found necessary to adjust the conditions to suit individuals, and in some cases the intervals were as little as two hours. In every case all lesions were thoroughly cleansed with soap and water before any dressing was applied. Peroxide of hydrogen was then applied; and this is highly recommended also for the painless removal of any dressing which becomes stuck and difficult to remove.

In burns of the first degree picric acid proved far the most satisfactory dressing, and in those of the second degree it also did very well. Though some of the patients passed darkened urine, no toxic or other ill effects were noted; and the extensive areas involved gave ample opportunity for absorption of the acid. The best results for the second stage were got, however, from the boracic baths; and for the third stage burns from the ichthyol ointment. There are certain other features of this series that are worth notice. Thus four patients developed duodenal ulceration, with severe hæmorrhage; all four recovered without any other treatment than the most rigid attention to diet. Notwithstanding the frequency of face burns, there was no case of asphyxia or of œdema of the glottis. No case terminated fatally, though in some three-quarters of the skin surface of the body was burnt, and even the slightest case had at least a fifth estimated to be burnt. In view of the extensive character of the burns and of the variety of local treatment employed, it may be conjectured that the nursing and general treatment was of the highest class; and the results obtained are most remarkable.

